# Decoding FGFR inhibitor sensitivity in cholangiocarcinoma with interpretable machine learning and cross-platform pharmacogenomic validation

**DOI:** 10.3389/fphar.2026.1807701

**Published:** 2026-04-30

**Authors:** Yading Xie, Honglei Li, Wujie Zhang, Shichao Sun, Junping Zhang, Lin Wang

**Affiliations:** Department 2 of Hepatobiliary Surgery, Handan First Hospital, Handan, Hebei, China

**Keywords:** biomarker discovery, cholangiocarcinoma, drug sensitivity, FGFR inhibitors, machine learning, pathway dependence, pharmacogenomics, transcriptomics

## Abstract

**Background:**

Fibroblast growth factor receptor (FGFR) inhibitors (FGFRis) benefit selected cholangiocarcinoma (CHOL) patients, yet responses remain heterogeneous and are not fully explained by canonical FGFR alterations. Clinically useful biomarkers are hindered by variable single-compound drug sensitivity readouts with limited concordance across FGFR-targeting agents and by optimistic bias arising from information leakage in pharmacogenomic modeling.

**Methods:**

We integrated large-scale pharmacogenomic screening with baseline transcriptomes to derive a pathway-level FGFRi sensitivity phenotype. Drug responses were z-normalized per compound and aggregated across an FGFR-targeting panel to generate a composite FGFRi score, with reliability assessed by drug–drug concordance and split-half reproducibility. Transcriptome-based predictors were trained using strict group-aware cross-validation with out-of-fold (OOF) evaluation; performance was quantified by Spearman correlation and tested by permutation. An interpretable transcriptional program was extracted from linear model coefficients and projected into CHOL cohorts. Portability was assessed via cross-platform concordance in an orthogonal resource (GDSC) using matched cell lines and via biological coherence across multiple CHOL patient datasets, including rank-based scoring and a reduced feature panel.

**Results:**

The composite FGFRi score was more stable than single-drug readouts and showed strong split-half reliability (median ρ = 0.63) without confounding by drug coverage. Baseline transcriptomes predicted FGFRi sensitivity under leakage-safe evaluation and yielded a compact, interpretable signature. In TCGA-CHOL, the signature mapped to structured tumor states and associated with FGFR-axis components, showing inverse correlations with FGFR1/2/3 and a positive correlation with KLB. In matched cell lines, PRISM-derived scores/signatures aligned with GDSC sensitivity for representative FGFR inhibitors. In external CHOL cohorts, rank-based scoring and leakage-controlled proxy-label models showed consistent performance across datasets, and a reduced 15-feature panel preserved concordance with the full signature.

**Conclusion:**

A multi-compound, pathway-level FGFRi phenotype coupled with leakage-safe transcriptomic modeling identifies a transferable, interpretable FGFRi-associated program. This framework improves reliability relative to single-drug biomarker discovery and supports practical, portable scoring for CHOL stratification. Prospective validation in FGFRi-treated CHOL cohorts is warranted.

## 1 Introduction

Cholangiocarcinoma (CCA) is an aggressive malignancy of the biliary tract with rising incidence in several regions and persistently poor outcomes despite advances in systemic therapy (Banales et al., 2020; Brindley et al., 2021; Valle et al., 2010; Oh et al., 2022). A major obstacle in CCA management is profound inter-patient heterogeneity at the molecular and phenotypic levels, which limits durable benefit from conventional chemotherapy and immunotherapy (Banales et al., 2020; Brindley et al., 2021; Oh et al., 2022). In this context, precision oncology strategies that exploit actionable oncogenic dependencies have become increasingly important for improving patient stratification and treatment selection (Banales et al., 2020; Brindley et al., 2021; Lamarca et al., 2020).

Among actionable targets, aberrant signaling in the fibroblast growth factor receptor (FGFR) pathway has emerged as a clinically relevant vulnerability, particularly in subsets of biliary tract cancers (Lamarca et al., 2020; Helsten et al., 2016; Krook et al., 2021). FGFR2 fusions/rearrangements and other FGFR-axis alterations provide a clear rationale for FGFR inhibitor (FGFRi) therapy, and multiple FGFR inhibitors have demonstrated meaningful activity in selected patients (Abou-Alfa et al., 2020; Javle et al., 2021; Goyal et al., 2023). Nevertheless, clinical responses remain heterogeneous: not all tumors harboring FGFR alterations respond, responses are often incomplete, and acquired resistance is common (Abou-Alfa et al., 2020; Javle et al., 2021; Goyal et al., 2023; Goyal et al., 2017). Conversely, a fraction of tumors without canonical FGFR alterations may exhibit functional FGFR pathway dependence and could potentially benefit from FGFR-targeted therapy but are not captured by alteration-based selection alone (Lamarca et al., 2020; Helsten et al., 2016; Krook et al., 2021). These observations suggest that genomics-only criteria do not fully represent the functional state of FGFR signaling or the broader cellular context that shapes drug response (Banales et al., 2020; Brindley et al., 2021; Lamarca et al., 2020).

A second, closely related challenge lies in translating preclinical pharmacogenomic knowledge into clinically actionable biomarkers (Haibe-Kains et al., 2013; Safikhani et al., 2016; Quevedo et al., 2020). Large-scale drug screening resources have enabled systematic mapping of drug response across hundreds of cancer cell lines, offering an opportunity to learn transcriptomic determinants of sensitivity and resistance (Corsello et al., 2020; Yang et al., 2013; Iorio et al., 2016). However, the FGFR inhibitor class illustrates a practical limitation of such efforts: compounds nominally targeting the same pathway can show only modest concordance in measured sensitivity due to differences in potency, selectivity, assay conditions, and context-specific polypharmacology (Haibe-Kains et al., 2013; Safikhani et al., 2016). As a result, biomarkers derived from a single compound may be unstable and fail to generalize across drugs or datasets (Haibe-Kains et al., 2013; Safikhani et al., 2016). Moreover, standard evaluation protocols in pharmacogenomics can be vulnerable to information leakage or optimistic bias if repeated measurements, shared biological entities, or preprocessing steps inadvertently transmit information from the test set into model training (Varma and Simon, 2006). These issues have contributed to inconsistent reproducibility and limited clinical translation of many proposed drug-response signatures (Haibe-Kains et al., 2013; Safikhani et al., 2016; Quevedo et al., 2020).

To address these barriers, we hypothesized that FGFR inhibitor response is better represented as a pathway-level phenotype rather than a single-drug endpoint, and that leakage-safe modeling of baseline transcriptomes can reveal transferable transcriptional programs associated with functional FGFR dependence (Haibe-Kains et al., 2013; Safikhani et al., 2016; Varma and Simon, 2006). Building on this hypothesis, we aggregated responses across an FGFR-targeting drug panel to derive a composite FGFRi sensitivity score with improved stability, explicitly designed to reduce single-compound noise and improve phenotype reliability (Haibe-Kains et al., 2013; Safikhani et al., 2016). We then trained and evaluated transcriptome-based prediction models using strict group-aware, out-of-fold procedures to prevent leakage and to obtain realistic generalization estimates (Varma and Simon, 2006). Beyond prediction, we aimed to extract an interpretable transcriptional signature that can be projected into tumor cohorts, enabling biological validation in patient data and facilitating practical translation into smaller gene panels.

Here, we present a framework that integrates (i) multi-compound pharmacogenomic phenotyping, (ii) leakage-safe machine learning on baseline transcriptomes, and (iii) cross-platform and cross-cohort validation, with a specific emphasis on cholangiocarcinoma relevance. We validate the inferred FGFRi-associated program in independent drug screening resources and multiple CHOL cohorts, including TCGA and GEO datasets, and we propose rank-based and reduced-panel implementations to improve portability across transcriptomic platforms (Farshidfar et al., 2017; Andersen et al., 2012; Chaisaingmongkol et al., 2017; Corsello et al., 2020; Yang et al., 2013). Collectively, this work seeks to provide a more consistent and clinically oriented framework for transcriptome-derived biomarkers that complement alteration-based FGFR selection and better capture the functional state underpinning FGFR inhibitor sensitivity in cholangiocarcinoma.

## 2 Methods

### 2.1 Study design and analytical overview

We developed a pathway-anchored, leakage-safe framework to model FGFR inhibitor (FGFRi) sensitivity by integrating pharmacogenomic drug screening with baseline transcriptomes. The workflow has three core steps: (i) phenotype engineering via multi-drug aggregation to construct a stable FGFRi sensitivity score, (ii) leakage-safe machine learning using group-aware cross-validation with out-of-fold (OOF) evaluation, and (iii) interpretability and translation through an FGFRi-associated transcriptional signature projected into cholangiocarcinoma (CHOL) cohorts and validated across independent drug-screen datasets.

### 2.2 Pharmacogenomic drug response data (PRISM) and FGFR inhibitor curation

#### 2.2.1 FGFR inhibitor selection

Drug response data were obtained from the PRISM Repurposing primary screen via the DepMap portal (accessed January 2026) (Corsello et al., 2020). The raw response $r_{i,d}$ was defined as the replicate-collapsed log2 fold-change in cell viability relative to DMSO control (single concentration 2.5 µM). Because more negative log2 fold-change indicates stronger growth inhibition, we oriented downstream standardized values so that larger $z_{i,d}$ (and thus larger composite FGFRi score) consistently indicates higher sensitivity across compounds. Compounds with insufficient measurement coverage across cell lines were excluded (coverage $c_{d}=n_{d}/N<0.8$ ). This threshold was chosen to ensure stable estimation of drug–drug concordance and to reduce missingness-driven variance in composite scores.

#### 2.2.2 Per-compound response standardization

Because drug response readouts can vary in dynamic range and measurement scale across compounds, responses were standardized within each drug across cell lines. For cell line i and drug d , the standardized response is Equation 1:

$$z_{i,d}=\frac{r_{i,d}-\mu_{d}}{\sigma_{d}}$$ (1)

where $r_{i,d}$ is the raw response, and $\mu_{d}$ and $\sigma_{d}$ are the mean and standard deviation across cell lines for drug d . This transformation preserves relative sensitivity ranking and mitigates compound-specific measurement scale effects. In PRISM, more negative log2 fold-change indicates higher sensitivity; therefore, after z-standardization we multiplied values by −1 so that larger $z_{i,d}$ (and thus larger $S_{i}$ ) consistently indicates higher sensitivity across compounds.

### 2.3 Multi-drug FGFRi sensitivity phenotype construction

#### 2.3.1 Rationale for aggregation

Single-drug sensitivity is inherently noisy and may reflect off-target activity or assay-specific variability. To approximate a pathway-level FGFR inhibition response phenotype, we integrated information across multiple FGFR-targeting compounds and treated the resulting composite as the primary modeling target.

#### 2.3.2 Unweighted composite FGFRi score

Let $D_{i}$ denote the set of curated FGFR inhibitors measured for cell line i . The unweighted composite FGFRi score is defined as:

$$S_{i}=\frac{1}{\left|D_{i}\right|}\sum_{d\in D_{i}}z_{i,d}$$ (2)

This aggregation reduces single-compound noise and provides a continuous phenotype capturing an FGFR-associated response axis.

#### 2.3.3 Weighted composite score

In addition to the unweighted score (Equation 2), we constructed a weighted composite phenotype to down-weight FGFR-targeting compounds that are (i) less concordant with the rest of the FGFR inhibitor panel and/or (ii) sparsely measured. Let $w_{d}\geq 0$ denote a drug-specific reliability weight. For each drug d, we computed its mean concordance with the remaining FGFR inhibitors using average pairwise Spearman correlation across cell lines with overlapping measurements Equation 3,

$${\bar{\rho }}_{d}=\frac{1}{\left|D\right|-1}\sum_{d^{\prime }\in D,d^{\prime }\neq d}\rho \left(d,d^{\prime }\right)$$ (3)

and its measurement completeness $c_{d}=n_{d}/N$ , where $n_{d}$ is the number of cell lines with observed response for drug d and N is the total number of profiled cell lines. We then defined Equation 4

$$c_{d}=\frac{n_{d}}{N},w_{d}=c_{d}\cdot \max\left(0,{\bar{\rho }}_{d}\right)$$ (4)

and normalized weights within each cell line’s available drug set $D_{i}$ . Weights were re-normalized within each $D_{i}$ such that $\sum_{d\in D_{i}}w_{d}=1$ (therefore $S_{i}^{\left(w\right)}=\sum_{d\in D_{i}}w_{d}z_{i,d}$ ). The weighted composite FGFRi score for cell line i is Equation 5

$$S_{i}^{\left(w\right)}=\frac{\sum_{d\in D_{i}}w_{d}z_{i,d}}{\sum_{d\in D_{i}}w_{d}}$$ (5)

here $D_{i}$ denotes the subset of curated FGFR inhibitors with non-missing measurements in cell line i . Unless otherwise stated, $S_{i}^{\left(w\right)}$ was used as the primary phenotype, while the unweighted score served as a sensitivity analysis.

#### 2.3.4 Split-half reliability of the phenotype

To quantify intrinsic reproducibility of the multi-drug phenotype and contextualize downstream predictability, we performed split-half analyses by randomly partitioning the FGFR drug set into two-halves, recomputing scores, and measuring their correlation. Reliability was summarized as Equation 6:

$$\rho_{\text{split}}=\text{Spearman}!\left(S_{i}^{\left(A\right)},S_{i}^{\left(B\right)}\right)$$ (6)

This provides an empirical upper bound on achievable predictive performance given phenotype noise.

### 2.4 Baseline transcriptomic profiles and preprocessing

Baseline gene expression profiles for cancer cell lines were obtained from the DepMap portal (Tsherniak et al., 2017; Ghandi et al., 2019). Expression matrices were log-transformed where appropriate and restricted to genes with sufficient variance across samples to remove near-invariant features. No outcome-driven feature selection was performed.

To prevent leakage, all feature scaling was performed within training folds only and then applied to held-out folds, ensuring that test-fold information did not influence preprocessing.

### 2.5 Leakage-safe model evaluation: GroupKFold and out-of-fold prediction

All predictive modeling used an outer GroupKFold cross-validation scheme with K = 5 folds, grouping by cell-line identity (Pedregosa et al., 2011). In the primary analysis, each cell line contributes a single baseline transcriptome and a single aggregated FGFRi phenotype, so GroupKFold is numerically equivalent to standard K-fold splitting; however, we report it explicitly to define the cell line as the unit of generalization and to ensure leakage-safe evaluation. Specifically, all preprocessing steps, including scaling, hyperparameter tuning, and coefficient estimation, were performed strictly within the training portion of each outer fold. Hyperparameters were selected using an inner GroupKFold procedure on the outer-training split only, and the optimized model was then applied to the held-out outer fold. Model evaluation relied exclusively on out-of-fold (OOF) predictions, defined as the concatenation of predictions generated on held-out folds across the outer GroupKFold splits. Let $k\left(i\right)\in \left\{1,\ldots ,K\right\}$ denote the fold index assigned to sample i . The OOF prediction for sample i is Equation 7

$${\hat{y}}_{i}=f^{\left(-k\left(i\right)\right)}\left(x_{i}\right)$$ (7)

where $f^{\left(-k\right)}$ is trained using only samples not in fold k . All reported metrics were computed exclusively from OOF predictions.

### 2.6 Machine-learning models, hyperparameter optimization, and selection

#### 2.6.1 Candidate models

We benchmarked multiple supervised learning algorithms reflecting distinct modeling assumptions: Ridge regression (L2-regularized linear), ElasticNet regression (L1/L2), support vector regression with radial basis function kernel (SVR-RBF), and random forest regression (Hoerl and Kennard, 1970; Zou and Hastie, 2005; Cortes and Vapnik, 1995; Breiman, 2001).

#### 2.6.2 Nested cross-validation for hyperparameter tuning

Hyperparameters were optimized using an inner GroupKFold with K_inner = 3 folds on the training split of each outer fold (Varma and Simon, 2006). Within each outer training fold, an inner GroupKFold procedure selected hyperparameters maximizing Spearman correlation, preventing overfitting due to tuning on the test fold.

#### 2.6.3 Dual-track strategy: performance vs. interpretability

Models were compared by OOF Spearman correlation. SVR-RBF achieved the highest predictive performance, whereas Ridge regression provided a stable and interpretable linear representation suitable for signature derivation under high-dimensional, correlated transcriptomic features. Accordingly, SVR-RBF was used to report maximal predictive accuracy, whereas Ridge was used as the primary linear model for derivation of the interpretable transcriptional program. ElasticNet was used separately to derive the reduced 15-feature panel for translation-oriented analyses.

### 2.7 Performance metrics, diagnostics, and statistical significance

#### 2.7.1 Primary performance metric

Predictive performance was quantified using Spearman rank correlation between observed FGFRi scores y and OOF predictions $\hat{y}$ Equation 8:

$$\rho_{\text{obs}}=\text{Spearman}!\left({\hat{y}}_{i},y_{i}\right)$$ (8)

Spearman correlation was chosen because the primary goal is to rank relative sensitivity rather than match absolute response magnitudes.

#### 2.7.2 Residual and calibration diagnostics

To assess systematic bias and error structure, we inspected residual distributions, residual-versus-predicted plots, and binned calibration curves computed from OOF predictions.

Statistical significance of the observed OOF performance was assessed by permutation testing (Phipson and Smyth, 2010). We performed B = 5,000 permutations by shuffling FGFRi scores across cell lines (i.e., permuting at the cell-line level), refit the full leakage-safe pipeline for each permutation, and recomputed the OOF Spearman correlation to obtain ${\left\{\rho_{b}\right\}}_{b=1}^{B}$ . Let $\rho_{\text{obs}}$ denote the observed OOF Spearman correlation. The one-sided empirical p-value is Equation 9

$$p=\frac{1+\sum_{b=1}^{B}I\left(\rho_{b}\geq \rho_{\text{obs}}\right)}{B+1}$$ (9)

### 2.8 Stratified analyses based on FGFR pathway context

Because FGFR inhibitor sensitivity is expected to depend on pathway activation, we performed mechanistically motivated stratified analyses based on baseline FGFR expression. FGFR-high subsets were defined as the top quartile of mean FGFR1–4 expression or the top quartile of individual FGFR family members. Importantly, stratification was applied *post hoc* to OOF predictions and was not used in training or hyperparameter selection, preserving leakage control. These analyses tested whether improved predictability aligns with FGFR pathway dependence rather than tissue-level confounding.

### 2.9 Derivation and analysis of interpretable transcriptional signatures

To enable biological interpretation and downstream projection to patient cohorts, we derived transcriptional signatures from Ridge regression models. No outcome-driven preselection of signature genes was performed. Instead, for each outer fold, ridge coefficients were extracted after within-fold feature standardization; coefficients were then averaged across folds to obtain final gene weights and to assess stability across splits. Genes with consistently large positive or negative coefficients and stable coefficient signs across folds were interpreted as components of an FGFR inhibitor response–associated transcriptional program (Supplementary Table S2).

Signature activity was subsequently summarized (i) using an “up–down” directional score for tumor cohorts and (ii) using ssGSEA-based activity scoring to improve cross-platform portability; rank-based scoring and an ElasticNet-derived reduced panel were further evaluated for portability and translation (as reflected in the downstream figure set) (Subramanian et al., 2005; Barbie et al., 2009; Hänz et al., 2013; Zou and Hastie, 2005). In GEO cohorts, gene-level ROC/AUC was computed using available-case evaluation (samples with non-missing expression for that gene and proxy labels; no imputation), so effective n may vary across genes.

## 3 Results

### 3.1 Overview of the leakage-safe learning and translation framework

**FIGURE 1:**
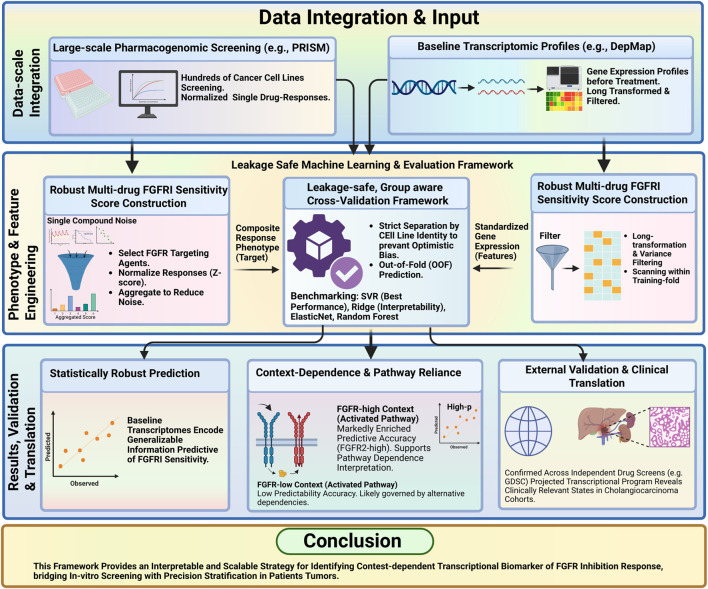
Study workflow for leakage-safe prediction of FGFR inhibitor sensitivity and translation to cholangiocarcinoma. Schematic of the analysis pipeline. Multi-drug FGFR inhibitor (FGFRi) responses from pharmacogenomic screening are standardized and aggregated into a composite FGFRi score, then predicted from baseline transcriptomes using group-aware, leakage-safe cross-validation with out-of-fold evaluation. The derived transcriptomic program is interpreted and validated across independent drug screens and projected to CHOL cohorts for translational assessment.

We integrated large-scale pharmacogenomic drug screening with baseline transcriptomic profiles to derive a stable, multi-compound fibroblast growth factor receptor inhibitor (FGFRi) sensitivity phenotype and to learn predictive transcriptomic features under a strict leakage-safe evaluation scheme (Figure 1).

### 3.2 Construction and quality control of a multi-drug FGFRi sensitivity score

**FIGURE 2:**
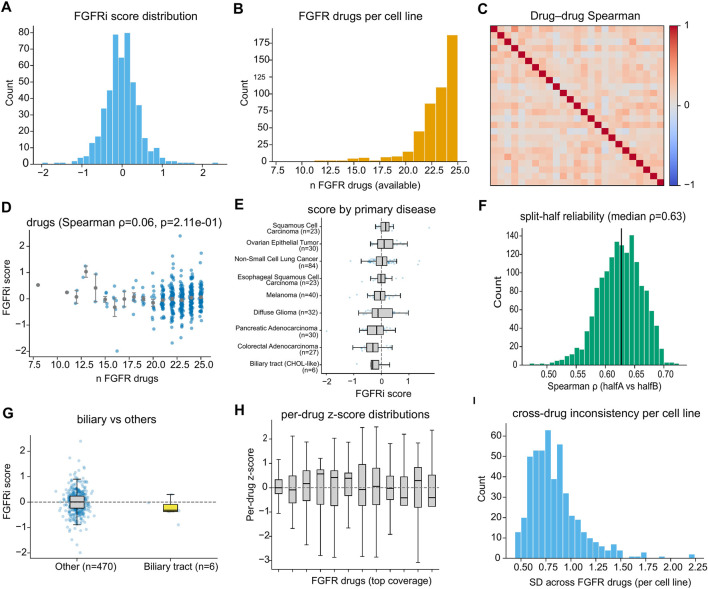
Multi-drug phenotyping defines a reliable composite FGFRi sensitivity score. **(A)** Distribution of the composite FGFRi score. **(B)** Drug coverage per cell line. **(C)** Modest pairwise concordance among FGFR-targeting drugs motivates aggregation. **(D)** Composite score is not driven by drug availability. **(E,G)** Lineage-specific distributions including biliary tract/CHOL-like lines. **(F)** Split-half analysis demonstrates reliability. **(H)** Per-drug standardized responses and **(I)** within-class variability across FGFR drugs. Higher composite FGFRi score indicates higher sensitivity (i.e., more negative PRISM log2 fold-change viability).

To reduce single-compound noise, we aggregated normalized responses across multiple FGFR-targeting agents into a composite FGFRi sensitivity score. The resulting score was approximately centered around zero with a unimodal distribution across screened cancer cell lines (Figure 2A). Drug coverage was high for most models, with the majority of cell lines having measurements for ∼20–25 FGFR-related compounds (Figure 2B). However, pairwise drug–drug concordance was generally modest (Figure 2C), consistent with compound-specific variability and reinforcing the need for aggregation.

Importantly, the composite FGFRi score was not confounded by the number of available FGFR drugs per cell line (Spearman ρ = 0.06, p = 2.11 × 10^−1^; Figure 2D), indicating that score magnitude was not simply driven by drug coverage. Across tumor lineages (analysis set, N = 476), the score showed lineage-dependent distributions, including biliary tract (CHOL-like) cell lines (n = 6) (Figure 2E). In a direct comparison to all other lineages (n = 470), biliary tract lines displayed a lower median FGFRi score (Figure 2G), although the small sample size warrants cautious interpretation.

We next evaluated score robustness. Split-half reliability analyses showed a median Spearman correlation of 0.63 between scores computed from two random halves of FGFR drugs (Figure 2F), supporting reproducibility of the aggregated phenotype. Per-drug z-score distributions further illustrated heterogeneous response behavior across compounds (Figure 2H), and the distribution of cross-drug variability (SD across FGFR drugs per cell line) demonstrated that a subset of models exhibited substantial inconsistency across FGFR-targeting agents (Figure 2I). Together, these results support the composite FGFRi score as a statistically more stable phenotype than any single-compound readout.

### 3.3 Baseline transcriptomes predict FGFRi sensitivity under group-aware, leakage-safe evaluation

**FIGURE 3:**
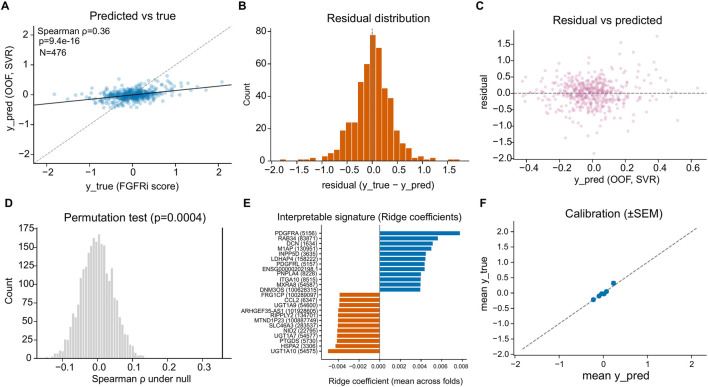
Baseline transcriptomes predict FGFRi sensitivity under leakage-safe evaluation and yield an interpretable signature. **(A)** Out-of-fold SVR predictions versus observed FGFRi scores. **(B,C)** Residual diagnostics. **(D)** Permutation test confirms significance. **(E)** Ridge coefficients define a compact positive/negative gene program. **(F)** Calibration of predicted versus observed scores across bins.

We trained models to predict the multi-drug FGFRi score from baseline transcriptomes using group-aware cross-validation with strict separation by cell line identity and out-of-fold (OOF) prediction (framework schematized in Figure 1). Support vector regression (SVR) produced statistically significant OOF predictive performance, with predicted vs. observed FGFRi scores correlated at Spearman ρ = 0.36 (p = 9.4 × 10^−16^; N = 476; Figure 3A). Residuals were centered near zero (Figure 3B) and did not show strong systematic dependence on predicted values (Figure 3C), consistent with limited gross bias across the prediction range.

A permutation test confirmed that the observed performance exceeded that expected under a null distribution (p = 4 × 10^−4^; Figure 3D). Calibration analysis showed agreement between binned mean predictions and mean observed values (±SEM), supporting the interpretability of predicted scores at the group level (Figure 3F).

To extract an interpretable transcriptional signature, we examined ridge regression coefficients averaged across folds. This analysis identified a compact set of positively and negatively weighted genes contributing to FGFRi score prediction (Figure 3E). Notably, the highest positive coefficients included PDGFRA, RAB34, and DCN, whereas negative coefficients included UGT1A10, HSPA2, and PTGDS (Figure 3E), establishing a candidate transcriptional program associated with FGFRi response. The fold-wise stability of the linear signature weights is summarized in Supplementary Table S2.

### 3.4 The FGFRi-associated transcriptional program maps onto clinically relevant states in cholangiocarcinoma

**FIGURE 4:**
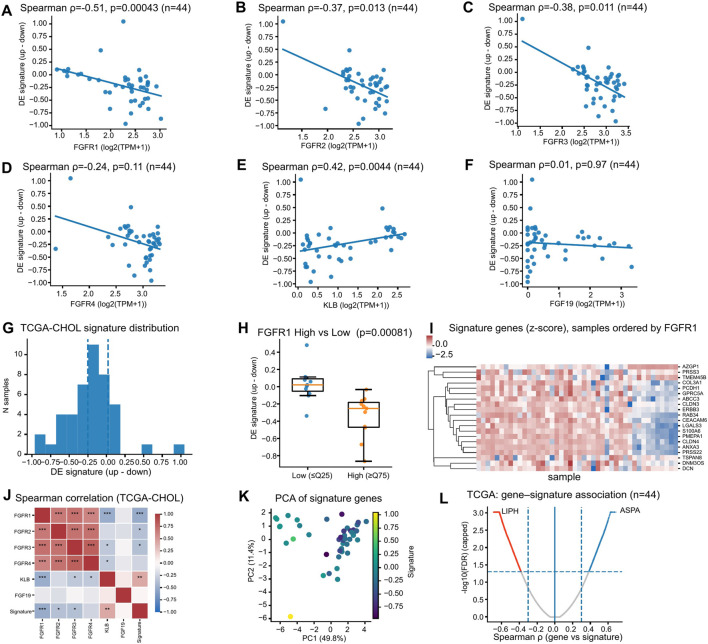
The FGFRi-associated program maps to structured tumor states in TCGA-CHOL. **(A–F)** Signature score correlates with FGFR-axis genes in TCGA-CHOL, showing inverse associations with FGFR1/2/3, a weaker non-significant association with FGFR4, a positive association with KLB, and minimal association with FGF19. **(G)** Distribution of the projected “up–down” signature score across TCGA-CHOL tumors. **(H,I)** FGFR1-high versus FGFR1-low stratification and heatmap of signature genes with samples ordered by FGFR1 expression. **(J,K)** Correlation structure among FGFR-axis genes and the signature score, and principal component analysis (PCA) of signature genes. **(L)** Transcriptome-wide gene–signature association highlights additional transcripts linked to the signature.

We next projected the inferred transcriptional program into cholangiocarcinoma tumor cohorts to assess whether the in vitro–derived signature captures meaningful biological variation in patient samples. In TCGA-CHOL (n = 44), an expression-based “up–down” signature score showed a broad distribution across tumors (Figure 4G). The signature was significantly inversely correlated with FGFR1 (Spearman ρ = −0.51, p = 4.3 × 10^−4^), FGFR2 (ρ = −0.37, p = 0.013), and FGFR3 (ρ = −0.38, p = 0.011) (Figures 4A–C), while being positively correlated with KLB (ρ = 0.42, p = 0.0044) (Figure 4E). FGFR4 showed a weaker and non-significant association (ρ = −0.24, p = 0.11; Figure 4D). In contrast, no association was observed with FGF19 (ρ = 0.01, p = 0.97; Figure 4F). These relationships were consistent with the correlation structure across FGFR-axis genes and the signature score within TCGA-CHOL (Figure 4J).

**FIGURE 5:**
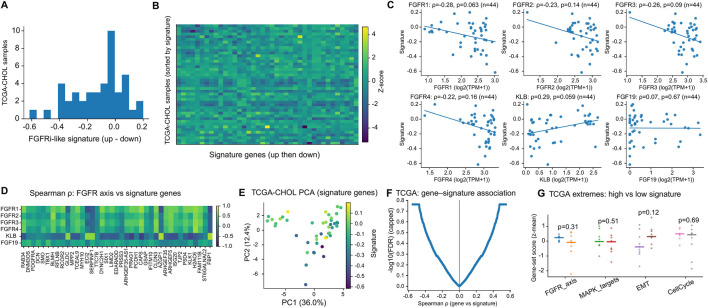
Alternative signature formulation and pathway-level comparisons in TCGA-CHOL. **(A,B)** Distribution/heatmap of an alternative FGFRi-like “up–down” score. **(C–F)** Weaker but directionally consistent associations with FGFR-axis genes and transcriptome-wide correlates. **(G)** Broad pathway scores (FGFR axis, MAPK, EMT, cell cycle) show limited separation between signature extremes in TCGA-CHOL.

Stratifying tumors by FGFR1 expression further supported a coordinated transcriptional shift: FGFR1-high samples (≥Q75) differed significantly from FGFR1-low samples (≤Q25) in signature score (p = 8.1 × 10^−4^; Figure 4H), and the corresponding heatmap revealed coherent expression patterns across signature genes when samples were ordered by FGFR1 (Figure 4I). Principal component analysis demonstrated that signature genes captured a major axis of transcriptional variation in TCGA-CHOL (PC1 = 49.8%; Figure 4K). A gene–signature association analysis highlighted additional transcripts strongly associated with the signature (e.g., LIPH and ASPA; Figure 4L). Functional enrichment of Figure 4L-associated genes further supported a broader tumor-state interpretation: positively associated genes were enriched for extracellular matrix organization and epithelial–mesenchymal remodeling programs, whereas negatively associated genes were enriched for mitochondrial respiration, oxidative phosphorylation, and ATP biosynthetic processes (Supplementary Figure S1). Together, these findings suggest that the projected program extends beyond FGFR transcripts and captures a coordinated cell-state axis rather than a single-gene surrogate. Alternative signature formulation and pathway-level comparisons in TCGA-CHOL are shown in Figure 5.

### 3.5 External validation in an independent drug screen supports generalizability

**FIGURE 6:**
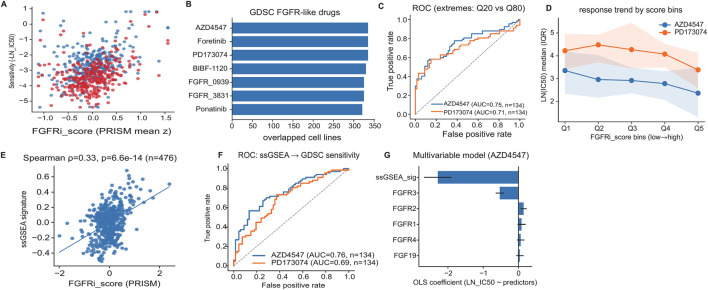
Cross-platform validation supports generalizability of the FGFRi phenotype and signature. **(A,B)** Cross-platform concordance: PRISM FGFRi score aligns with GDSC drug sensitivity in matched cell lines. **(C,D)** Extreme-group ROC and binned trends for representative FGFR inhibitors (AZD4547, PD173074; ≤Q20 vs. ≥Q80; N = 134 matched cell lines). **(E,F)** ssGSEA signature tracks FGFRi score and predicts GDSC sensitivity extremes (≤ Q20 vs. ≥ Q80; N = 134). **(G)** Multivariable modeling shows the signature adds information beyond individual FGFR-axis transcripts. Higher FGFRi score indicates higher sensitivity (more negative PRISM log2 fold-change; lower GDSC ln (IC50)).

To assess cross-platform concordance, we evaluated whether the PRISM-derived FGFRi score aligned with drug sensitivity profiles measured in the orthogonal GDSC screening resource in matched cell lines across multiple FGFR-like compounds (Figure 6B). In matched cell lines, the PRISM FGFRi score tracked GDSC drug sensitivity for representative FGFR inhibitors (Figure 6A). Because this analysis compares two assay platforms on the same biological entities, it primarily evaluates assay/platform portability rather than generalization to unseen cell lines. When classifying extreme responders (≤Q20 vs. ≥Q80) using the FGFRi score, ROC/AUC was computed on the extreme subsets only (n_ext ≈ 0.4×N; N = 134 matched cell lines for each compound), yielding AUC = 0.75 for AZD4547 and AUC = 0.71 for PD173074 (Figure 6C). Consistent with this, median ln (IC50) decreased monotonically across increasing FGFRi score bins for both compounds, demonstrating a graded response trend rather than a purely dichotomous separation (Figure 6D).

We then examined whether the transcriptomic signature derived from baseline expression retained predictive value across datasets. A signature summary computed by ssGSEA correlated with the FGFRi score in PRISM (Spearman ρ = 0.33, p = 6.6 × 10^−14^; n = 476; Figure 6E). Using ssGSEA scores alone to predict GDSC sensitivity in extreme groups yielded AUC = 0.76 for AZD4547 and AUC = 0.69 for PD173074 (N = 134 matched cell lines per compound; Figure 6F), supporting portability of the transcriptional program across screening platforms.

In a multivariable model of AZD4547 sensitivity (ln (IC50) as the outcome), the ssGSEA signature displayed the largest coefficient magnitude relative to individual FGFR family transcripts and FGF19 (Figure 6G), consistent with the signature capturing information not reducible to single-gene surrogates. A quantitative summary of cross-platform continuous agreement, extreme-group discrimination, exact P values, and confidence intervals is provided in Supplementary Table S1.

### 3.6 Cross-cohort consistency in cholangiocarcinoma datasets and a compact panel for translation

**FIGURE 7:**
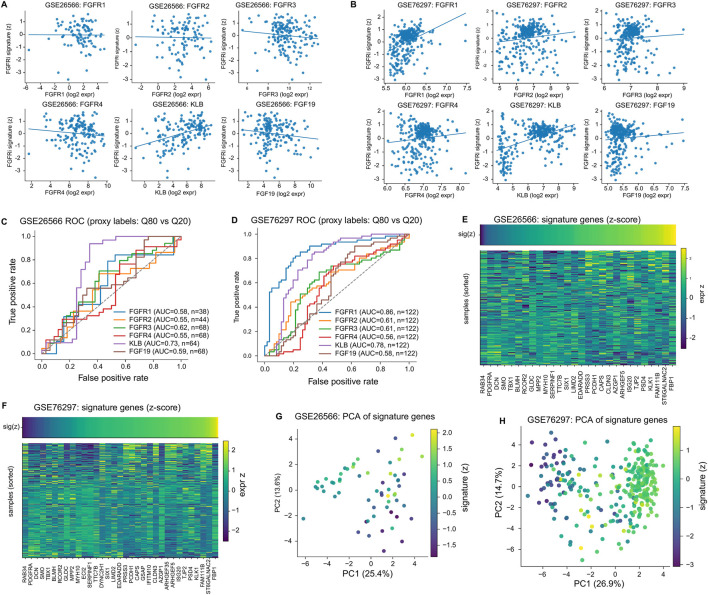
Consistency of the FGFRi signature across independent CHOL cohorts. **(A,B)** Signature–FGFR axis relationships in GSE26566 and GSE76297. **(C,D)** ROC analyses in GSE26566/GSE76297 using proxy labels defined by extreme quantiles (≤Q20 vs. ≥Q80); N indicates the total number of samples with available expression and proxy labels for the corresponding gene (available-case; no imputation), and ROC/AUC was computed on the extreme subsets; therefore, effective sample counts can vary across genes. **(E–H)** Heatmaps and PCA show coherent signature-driven structure across both CHOL cohorts.

We further assessed signature behavior across two independent cholangiocarcinoma transcriptomic cohorts (GSE26566 and GSE76297). Across these cohorts, the FGFRi signature score showed cohort-specific but structured relationships with FGFR-axis components (Figures 7A,B), and the signature genes formed coherent expression patterns in heatmaps and low-dimensional embeddings (Figures 7E–H). In proxy ROC analyses (Q80 vs. Q20), several single-gene markers such as KLB and FGFR1 showed relatively strong discrimination, particularly in GSE76297 (e.g., FGFR1 AUC = 0.86; KLB AUC = 0.78; Figure 7D) and more modestly in GSE26566 (KLB AUC = 0.73; Figure 7C), emphasizing both reproducibility and cross-cohort heterogeneity. Because gene expression availability differs after platform-specific probe-to-gene mapping, ROC/AUC was computed using available-case evaluation (no imputation), so the effective sample size varies by gene; proxy labels were defined by extreme quantiles (≤Q20 vs. ≥Q80) and ROC/AUC was computed on the corresponding extreme subsets (Figures 7C,D).

**FIGURE 8:**
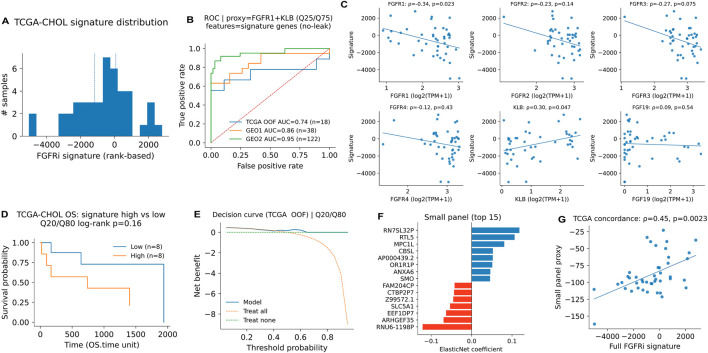
Rank-based scoring and reduced panels improve portability and enable leakage-controlled proxy prediction. **(A)** Rank-based signature distribution in TCGA-CHOL. **(B)** Leakage-controlled ROC performance using proxy labels defined by FGFR1+KLB extremes (≤Q25 vs. ≥Q75). For TCGA, n = 18 denotes the samples included in the OOF proxy-label analysis; for external GEO analyses, N denotes the number of samples with available labels and expression. ROC/AUC was computed on the corresponding extreme subsets. **(C)** Associations with FGFR1 and KLB. **(D,E)** Exploratory OS Kaplan–Meier and decision curve analyses; these analyses are hypothesis-generating only and should not be interpreted as evidence of clinical benefit. **(F,G)** Elastic net–derived small panel and its concordance with the full signature.

To support platform-robust application, we implemented a rank-based version of the FGFRi signature in TCGA-CHOL (Figure 8A). This rank-based signature was significantly inversely correlated with FGFR1 expression (ρ = −0.34, p = 0.023) and positively correlated with KLB (ρ = 0.30, p = 0.047) (Figure 8C). Exact P values and confidence intervals for the key TCGA-CHOL correlations of both the main projected signature and the rank-based signature are summarized in Supplementary Table S4. In a leakage-controlled evaluation using a proxy label defined by FGFR1+KLB extremes (≤Q25 vs. ≥Q75), models using signature genes achieved AUC = 0.74 in TCGA out-of-fold prediction (n = 18 samples in the OOF proxy-label analysis) and maintained strong performance in external GEO cohorts (GSE26566: AUC = 0.86, N = 38; GSE76297: AUC = 0.95, N = 122), where N denotes the number of samples with available labels and expression; in all cases, ROC/AUC was computed on the corresponding extreme subsets (Figure 8B).

Finally, we performed exploratory, hypothesis-generating analyses in TCGA-CHOL. Comparing overall survival between signature-high and signature-low tumors (Q80 vs. Q20; n = 8 per group) showed no statistically significant difference (log-rank p = 0.16; Figure 8D). An exploratory decision curve analysis is shown in Figure 8E; these results are hypothesis-generating only and should not be interpreted as evidence of clinical benefit, particularly given the very small group size and the absence of treatment-response endpoints. To facilitate future assay development, an elastic net–selected compact panel (top 15 features) was derived (Figure 8F) and showed significant concordance with the full signature in TCGA, indicating that a reduced gene set can partially recapitulate the broader program.

## 4 Discussion

FGFR inhibitors have reshaped precision oncology, yet clinical benefit remains heterogeneous even among tumors with canonical FGFR alterations (Lamarca et al., 2020; Abou-Alfa et al., 2020; Javle et al., 2021; Goyal et al., 2023). A key gap is that genomics alone often fails to capture functional FGFR pathway dependence (Banales et al., 2020; Brindley et al., 2021; Lamarca et al., 2020). In this work, we addressed this gap by engineering a pathway-level drug response phenotype and coupling it to leakage-safe transcriptome-based modeling, then translating the learned program to cholangiocarcinoma cohorts and independent screening resources. Our findings support three overarching conclusions: (i) multi-drug phenotype engineering yields a more reliable FGFR inhibition sensitivity axis than single-compound readouts, (ii) baseline transcriptional state encodes a real—though incomplete—component of FGFRi response potential under rigorous evaluation, and (iii) this signal is strongly context-dependent and transferable across platforms and CHOL patient cohorts, providing a tractable route toward transcriptomic biomarkers that complement genomic selection (Haibe-Kains et al., 2013; Safikhani et al., 2016; Varma and Simon, 2006).

A central methodological choice was to aggregate responses across multiple FGFR-targeting agents into a composite FGFRi score. This decision is empirically justified by the modest drug–drug concordance within the FGFR inhibitor panel and the heterogeneous per-drug response distributions, which together indicate that single-agent measurements are strongly influenced by compound-specific variability (Haibe-Kains et al., 2013; Safikhani et al., 2016). By contrast, the composite score demonstrated meaningful intrinsic reliability and was not materially confounded by drug coverage, supporting its interpretation as a denoised, pathway-level phenotype that more closely tracks a latent FGFR dependence axis (Haibe-Kains et al., 2013; Safikhani et al., 2016). Importantly, this score should be viewed as a relative sensitivity axis for ranking and prioritization, rather than as a clinically calibrated responder threshold. This approach also aligns with the biological reality that target class response is rarely “one drug = one truth,” and it provides a principled foundation for biomarker discovery that is less sensitive to idiosyncrasies of any specific inhibitor (Haibe-Kains et al., 2013; Safikhani et al., 2016).

Using strict group-aware cross-validation and out-of-fold predictions, we observed statistically significant predictive performance (Varma and Simon, 2006). Residual and calibration diagnostics further suggest that the signal is not dominated by extreme points or systematic bias. These results imply that baseline transcriptional state contains consistent information relevant to FGFR inhibition response, consistent with a model in which cell-state programs modulate pathway wiring, feedback resilience, and adaptive capacity.

At the same time, the magnitude of association is moderate—appropriately so. Drug response is governed by multiple determinants that are only partially captured by baseline mRNA, including gene fusions and copy-number changes, post-translational signaling states, and lineage-dependent epigenetic context (Helsten et al., 2016; Krook et al., 2021; Goyal et al., 2017). The split-half reproducibility of the phenotype itself also places an empirical ceiling on achievable prediction from any input modality; therefore, the observed performance should be interpreted as evidence of transferable signal rather than a claim of deterministic predictability.

A key scientific question is whether transcriptome-based predictability reflects genuine FGFR pathway reliance or merely correlates with lineage structure. Our framework explicitly emphasizes context dependence, and the results support that view: predictive accuracy is enriched in FGFR-activated contexts, with particular strength in FGFR2-high settings as summarized in the schematic. Conceptually, this is the expected behavior of a pathway-dependence biomarker: when tumors are plausibly “on-target” FGFR-driven, baseline transcriptomic state is more informative for FGFRi response; conversely, FGFR-low contexts likely reflect alternative dependencies and thus lower predictability. This implies the correct translational framing is enrichment rather than pan-cancer universal prediction: the signature/model is most valuable for prioritizing patients or models within FGFR-activated subsets where FGFR inhibitors are mechanistically relevant (Lamarca et al., 2020; Helsten et al., 2016; Krook et al., 2021).

Beyond prediction, we aimed for interpretability. Ridge coefficients highlighted a set of genes with stable positive/negative weights, enabling biological hypothesis generation and downstream projection. Importantly, translation to TCGA-CHOL demonstrated that the derived signature captures structured tumor states and is not simply a proxy for any single FGFR transcript: the signature showed significant inverse correlations with FGFR1/2/3 and a positive correlation with KLB, while FGF19 showed no association in this cohort (Farshidfar et al., 2017). The coherent heatmap structure and PCA separation further support that the signature reflects a broader transcriptional program (Farshidfar et al., 2017). The positive association with KLB is intriguing and may reflect a broader FGFR-axis contextual state or hepatobiliary competence program; however, our data do not support direct inference of canonical FGF19–FGFR4 activation, because FGFR4 showed only a weaker, non-significant association and FGF19 showed no association in TCGA-CHOL.

The inverse correlations with FGFR1/2/3 and the enrichment of mesenchymal/stromal-associated genes (e.g., PDGFRA, DCN) raise the possibility that the signature partly reflects a cell-state axis (e.g., EMT-like programs) rather than a direct readout of FGFR transcript abundance. Consistent with this interpretation, functional enrichment of Figure 4L-associated genes showed that positively associated genes were enriched for extracellular matrix organization and EMT-related remodeling, whereas negatively associated genes were enriched for mitochondrial respiration, oxidative phosphorylation, and ATP biosynthetic processes (Supplementary Figure S1). Importantly, the program was learned from cancer cell lines, which lack tumor microenvironment components, suggesting that these genes may capture tumor-intrinsic state variation that modulates FGFRi sensitivity. Nevertheless, bulk TCGA-CHOL projections can be influenced by tumor purity and stromal content; therefore, the signature should be interpreted as a tumor-state–linked program that aligns with FGFR-axis context, rather than a direct mechanistic assay of FGFR signaling.

From a biomarker standpoint, this is an important distinction. Clinical FGFR selection often relies on discrete genomic events, yet response heterogeneity persists (Abou-Alfa et al., 2020; Javle et al., 2021; Goyal et al., 2023; Goyal et al., 2017). A transcriptional program that captures pathway competence and cellular context could complement alteration-based selection by identifying states more likely to be functionally FGFR-dependent or more resistant due to alternative programs (Lamarca et al., 2020; Krook et al., 2021; Goyal et al., 2017).

A frequent failure mode in pharmacogenomic modeling is screen-specific overfitting (Haibe-Kains et al., 2013; Safikhani et al., 2016; Varma and Simon, 2006). Here, multiple analyses support cross-platform generalizability. The PRISM-derived FGFRi score aligned with GDSC drug sensitivity for FGFR-like compounds, with meaningful discrimination in extreme-group analyses and monotonic trends across FGFRi score bins (Corsello et al., 2020; Yang et al., 2013). An ssGSEA summary of the signature correlated with FGFRi score and predicted GDSC sensitivity, suggesting portability of the learned program (Subramanian et al., 2005; Barbie et al., 2009; Hänz et al., 2013; Corsello et al., 2020; Yang et al., 2013). Moreover, in multivariable modeling of AZD4547 sensitivity, the ssGSEA signature contributed a larger coefficient magnitude than individual FGFR-axis transcripts, consistent with the signature capturing information beyond single-gene readouts (Subramanian et al., 2005; Barbie et al., 2009; Hänz et al., 2013).

For deployment across heterogeneous transcriptomic platforms, we further implemented a rank-based signature formulation that maintains associations with FGFR1 and KLB and achieved strong AUCs in TCGA out-of-fold and external GEO cohorts using proxy labels (Farshidfar et al., 2017; Andersen et al., 2012; Chaisaingmongkol et al., 2017). The reduced “small panel” and its concordance with the full signature provide a practical pathway toward assay development where limited-gene panels are often required (Zou and Hastie, 2005).

### 4.1 Limitations

Several limitations should be recognized. First, the CHOL sample sizes are modest in TCGA and the biliary tract cell-line subset is very small, limiting lineage-specific conclusions and emphasizing the need for CHOL-focused model systems and larger cohorts. Accordingly, the FGFRi-associated transcriptional program should be interpreted as a pan-cancer–derived, CHOL-relevant program that can be projected to cholangiocarcinoma, rather than a CHOL-specific model trained within a CHOL-only system. In sensitivity analyses, leave-biliary-out re-estimation showed near-identical model coefficients, limited changes in biliary-line predictions, and highly concordant projected TCGA-CHOL scores after exclusion of biliary tract lines (Supplementary Figure S2), supporting the stability of the learned program. Nevertheless, definitive CHOL-specific refinement will require larger cholangiocarcinoma-focused pharmacogenomic datasets. Second, the patient-cohort analyses establish biological coherence and FGFR-axis alignment rather than direct prediction of FGFRi clinical response; definitive clinical utility requires cohorts with treatment and response endpoints (Abou-Alfa et al., 2020; Javle et al., 2021; Goyal et al., 2023). Defining treatment-decision cutoffs will require FGFRi-treated cholangiocarcinoma cohorts with direct clinical response endpoints. Third, while transcriptomes carry predictive signal, multi-omics integration will likely improve mechanistic specificity and performance ceilings (Helsten et al., 2016; Krook et al., 2021).

## 5 Conclusion

In this study, we developed a leakage-safe and interpretable framework to quantify and predict FGFR inhibitor sensitivity by integrating large-scale pharmacogenomic screening with baseline transcriptomic profiles. By constructing a multi-drug composite FGFRi score, we reduced single-compound noise and defined a pathway-level response phenotype with strong internal reliability. Using group-aware cross-validation, we demonstrated that baseline gene expression encodes consistent predictive signal for FGFRi sensitivity and derived an interpretable transcriptional program associated with response. This signature generalized across independent datasets and platforms, aligning with FGFR inhibitor sensitivity in external screening resources and capturing coherent tumor states and consistent FGFR-axis relationships in cholangiocarcinoma cohorts. A rank-based formulation and reduced gene panel further enhanced portability and feasibility for downstream assay development. Together, these findings support a model in which FGFR inhibitor response is partly determined by baseline transcriptional state and is most informatively assessed at the pathway or program level rather than through single-gene surrogates, providing a scalable foundation for developing transcriptome-derived biomarkers that may complement genomic selection in FGFR-targeted therapy, pending validation in FGFRi-treated cholangiocarcinoma cohorts.

## Data Availability

Publicly available datasets were analyzed in this study. This data can be found here: The datasets analyzed in this study are publicly available. Transcriptomic and drug response data can be found in the DepMap portal (https://depmap.org/portal/) and the GDSC database (https://www.cancerrxgene.org/). Patient transcriptomic datasets were obtained from The Cancer Genome Atlas (TCGA) via the GDC Data Portal and the NCBI Gene Expression Omnibus (GEO) under accession numbers GSE26566 and GSE76297.
