# Supplementary material for: Decoding FGFR inhibitor sensitivity in cholangiocarcinoma with interpretable machine learning and cross-platform pharmacogenomic validation
Source: Front Pharmacol. 2026 Apr 30;17:1807701. doi: 10.3389/fphar.2026.1807701 (PMC13171567; doi:10.3389/fphar.2026.1807701)
Supplement: Supplementary file 1 [file DataSheet2.pdf]

Supplementary Figures

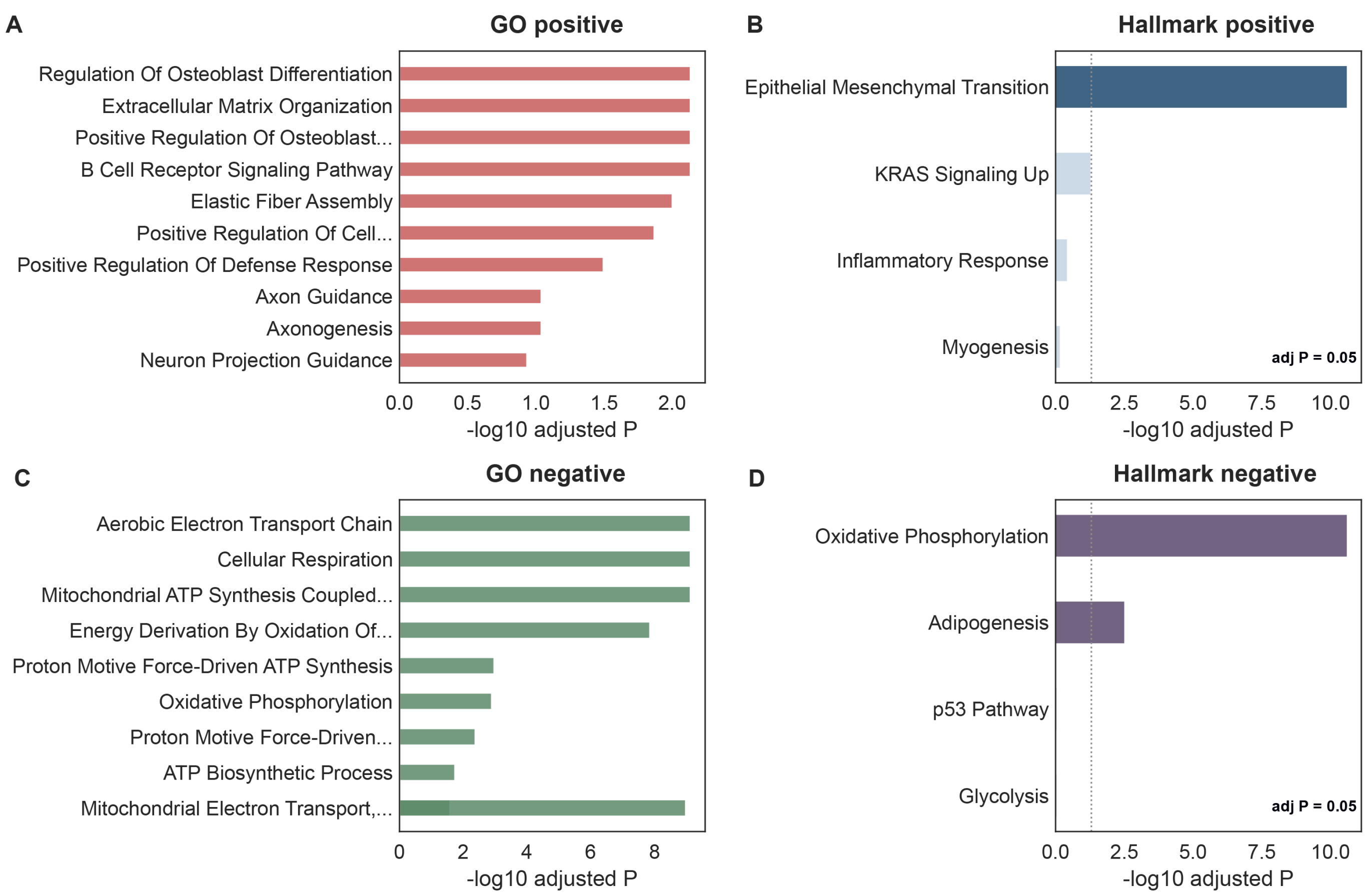

Supplementary Figure S1. GO and Hallmark enrichment of genes associated with the Figure 4L signature. Gene ontology (GO) and Hallmark enrichment analyses were performed separately for genes positively and negatively associated with the projected signature shown in Figure 4L. **(A)** GO enrichment for positively associated genes. **(B)** Hallmark enrichment for positively associated genes. **(C)** GO enrichment for negatively associated genes. **(D)** Hallmark enrichment for negatively associated genes. Positive-associated genes were enriched for extracellular matrix organization, epithelial–mesenchymal transition (EMT)-related remodeling, and related developmental or stress-response processes, whereas negative-associated genes were enriched for mitochondrial respiration, oxidative phosphorylation, and ATP biosynthetic programs. Bar lengths indicate −log10 adjusted P values. In the Hallmark panels, the dotted vertical line marks the adjusted $P = 0.05$ threshold.

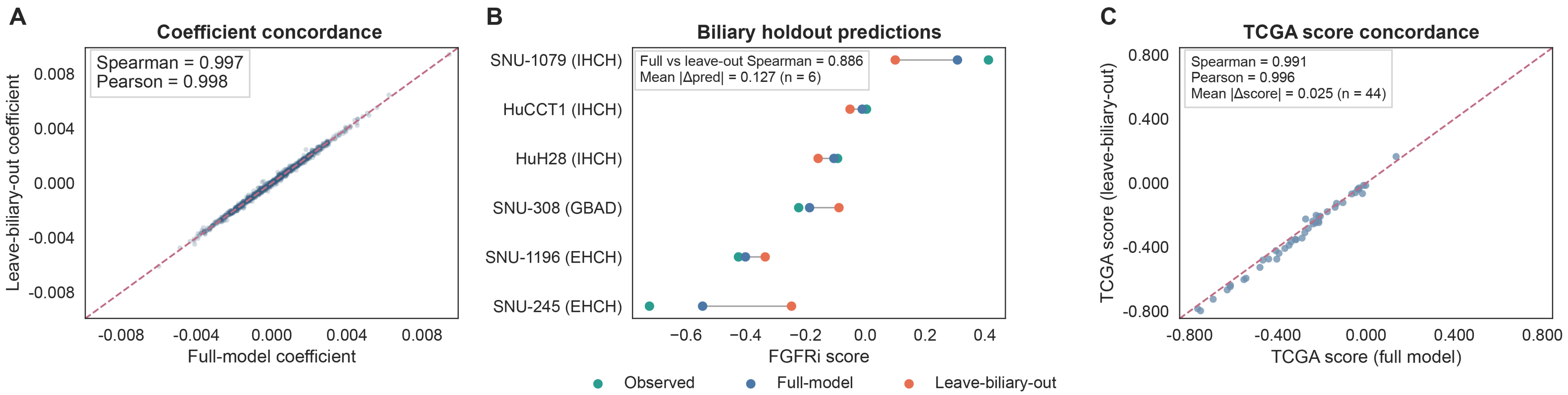

Supplementary Figure S2. Leave-biliary-out sensitivity analysis of signature stability, biliary-line predictions, and TCGA projection concordance. To assess whether the small biliary tract cell-line subset disproportionately influenced signature derivation or downstream cholangiocarcinoma projection, the full model was re-estimated after excluding all biliary tract cell lines. **(A)** Concordance of shared gene coefficients between the full model and the leave-biliary-out model. **(B)** Observed FGFRi scores, full-model predictions, and leave-biliary-out predictions for the six biliary tract cell lines. **(C)** Concordance between projected TCGA-CHOL signature scores obtained from the full model and the leave-biliary-out model. Together, these analyses show that both the inferred transcriptional program and its projection to TCGA-CHOL remained highly stable after exclusion of biliary tract lines.
